# The Genetics of Speciation by Reinforcement

**DOI:** 10.1371/journal.pbio.0020416

**Published:** 2004-11-23

**Authors:** Daniel Ortiz-Barrientos, Brian A Counterman, Mohamed A. F Noor

**Affiliations:** **1**Department of Biological Sciences, Louisiana State UniversityBaton Rouge, LouisianaUnited States of America

## Abstract

Reinforcement occurs when natural selection strengthens behavioral discrimination to prevent costly interspecies matings, such as when matings produce sterile hybrids. This evolutionary process can complete speciation, thereby providing a direct link between Darwin's theory of natural selection and the origin of new species. Here, by examining a case of speciation by reinforcement in *Drosophila,* we present the first high-resolution genetic study of variation within species for female mating discrimination that is enhanced by natural selection. We show that reinforced mating discrimination is inherited as a dominant trait, exhibits variability within species, and may be influenced by a known set of candidate genes involved in olfaction. Our results show that the genetics of reinforced mating discrimination is different from the genetics of mating discrimination between species, suggesting that overall mating discrimination might be a composite phenomenon, which in *Drosophila* could involve both auditory and olfactory cues. Examining the genetics of reinforcement provides a unique opportunity for both understanding the origin of new species in the face of gene flow and identifying the genetic basis of adaptive female species preferences, two major gaps in our understanding of speciation.

## Abstract 2

Mating discrimination between two species of Drosophila is more pronounced where they hybridize and genes related to odor appear responsible for this "reinforcement," thus providing insights into the genetics of speciation

## Introduction

During reinforcement, mating discrimination is strengthened by natural selection in response to maladaptive hybridization between closely related taxa (Dobzhansky 1940; Fisher 1958). Although reinforcement was a contentious issue in the past (Butlin 1989; Howard 1993; Noor 1999), recent theoretical work has identified the most favorable conditions for its existence (Liou and Price 1994; Kelly and Noor 1996; Servedio and Kirkpatrick 1997; Kirkpatrick and Servedio 1999; Servedio 2000), and empirical data have provided potential examples of its occurrence in nature (Noor 1995; Sæaetre et al. 1997; Rundle and Schluter 1998; Nosil et al. 2003).

Theoretical work on reinforcement shows that reproductive isolation may be strengthened when either the same (one) or different (two) alleles conferring mating discrimination spread in the emerging species (Felsenstein 1981). In two*-*allele models, alleles conferring mating discrimination spread if they become genetically correlated with alleles reducing hybrid fitness. However, the evolution of such a correlation is opposed by recombination because alleles conferring discrimination in a given species do not confer discrimination in the other species. Consequently, two*-*allele models require either very strong selection, or tight linkage (e.g., physical or via chromosomal rearrangements) between alleles conferring mating discrimination and alleles reducing hybrid fitness (Kirkpatrick and Ravigné 2002). In contrast, one-allele models are not opposed by recombination because alleles conferring mating discrimination reduce hybridization in the genetic background of both species (Kelly and Noor 1996; Servedio 2000) and so may be more conducive to reinforcement. Unfortunately, empirical data concerning these two models of speciation are lacking (see Ortíz-Barrientos et al. 2002; Servedio and Noor 2003).

In addition to providing fundamental information for theoretical models, discerning the genetics of reinforcement will also develop our understanding of both the physiological basis of and forces governing changes in female preference. Furthermore, because the strengthening of female preference is driven by natural selection, the genetics of reinforcement will provide unique insights into the genetics of adaptation, another unsettled issue in evolutionary biology. Finally, high-resolution genetic studies of reinforcement can identify candidate speciation genes with effects on mating discrimination, information almost nonexistent in speciation studies (but see, Ritchie and Noor 2004). Here, we address these many fundamental issues in speciation by examining a case of reinforcement in *Drosophila* and present for the first time a high-resolution genetic study of variation within species in female mating discrimination, including a set of candidate reinforcement genes and a discussion of the evolutionary implications of our findings.

The North American fruitflies Drosophila pseudoobscura and D. persimilis hybridize in nature and produce sterile male hybrids. While D. pseudoobscura occurs alone in non-coastal western United States and Central America, the two species co-occur in California and the Pacific Northwest. Males court females from both species indiscriminately (Noor 1996), but females mate preferentially with individuals from the same species. The strength of this discrimination is not homogeneous across the species' geographic range: in a previous study, Noor (1995) showed that D. pseudoobscura females derived from populations where D. persimilis was absent exhibited weak mating discrimination (hereafter, “basal mating discrimination”) while females derived from populations where D. persimilis is present exhibited strong mating discrimination (hereafter “reinforced mating discrimination”). This difference in mating discrimination is likely the evolutionary consequence of maladaptive hybridization where the two species coexist: reinforcement has strengthened mating discrimination in the D. pseudoobscura populations co-occurring with D. persimilis. These observations and the recent completion of the genome sequence of D. pseudoobscura (BCM-HGSC 2004) make these species an ideal system to genetically dissect the enhancement of mating discrimination in sympatry.

Although the genetics of reinforcement has not been studied in *D. pseudoobscura,* or in any system, the genetic basis of other traits contributing to the species' reproductive isolation (i.e., hybrid sterility and basal mating discrimination) is known in detail. All traits contributing to reproductive isolation between D. pseudoobscura and *D. persimilis,* including traits for basal discrimination, map primarily or exclusively to regions bearing fixed chromosomal inversion differences between the species (Noor et al. 2001a, 2001b). This result is consistent with a two*-*allele model of speciation in which the reduction in recombination between alleles for hybrid unfitness (i.e., hybrid sterility) and mating discrimination creates the necessary genetic correlations to advance divergence in the presence of gene flow. However, we do not know whether the genetic basis of reinforced mating discrimination corresponds to this picture, and specifically, whether chromosomal inversions are fundamental to this process. Comparing these genetic architectures will provide the most comprehensive view yet on the genetics of mating discrimination contributing to the formation of new species in the face of interspecies gene flow.

## Results

### Female Discrimination Is Dominant and Reinforced in Sympatry

Table 1 shows that D. pseudoobscura females derived from sympatry (with D. persimilis) exhibited stronger mating discrimination against D. persimilis males than did D. pseudoobscura females derived from allopatry. This pattern holds for both inbred and outbred lines. Also, our data show that both sympatric-derived lines and allopatric-derived lines vary considerably in their degree of discrimination (*p* < 0.001 for sympatric inbred lines, and *p* = 0.0006 for allopatric inbred lines), suggesting some within-population variation in female mating discrimination, both basal and reinforced.

**Table 1:**
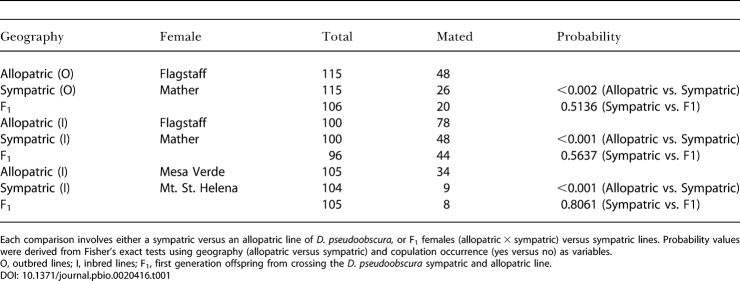
Matings of D. persimilis Males to D. pseudoobscura Females Derived Sympatry or Allopatry

Each comparison involves either a sympatric versus an allopatric line of *D. pseudoobscura,* or F_1_ females (allopatric × sympatric) versus sympatric lines. Probability values were derived from Fisher's exact tests using geography (allopatric versus sympatric) and copulation occurrence (yes versus no) as variables

O, outbred lines; I, inbred lines; F_1_, first generation offspring from crossing the D. pseudoobscura sympatric and allopatric line

10.1371/journal.pbio.0020416.t001

Apparent reinforced mating discrimination could result from behavioral differences in D. persimilis males when exposed to sympatric or allopatric D. pseudoobscura females. To exclude this possibility, we measured the copulation latency and number of attempted copulations by D. persimilis males towards D. pseudoobscura females derived from sympatry or allopatry, and found no significant differences between groups (copulation latency, *p* = 0. 736, *n* = 138; attempted copulations, *p* = 0. 937, *n* = 110). Finally, we investigated the mode of inheritance of the phenotype and observed that F_1_ females from crosses between sympatric and allopatric flies discriminated as strongly as their sympatric parent, suggesting that reinforced female mating discrimination is inherited as a dominant trait in both inbred and outbred lines (see Table 1). This F_1_ female mating discrimination is restricted to pairings with D. persimilis males, as F_1_ females mate readily with conspecifics (data not shown). Taken together, these results suggest that reinforced mating discrimination in D. pseudoobscura is exclusive to females derived from areas of sympatry with D. persimilis, is inherited as a dominant trait, and is not markedly affected by inbreeding.

### Within-Species Variation in Reinforced Female Mating Discrimination

**Figure 1:**
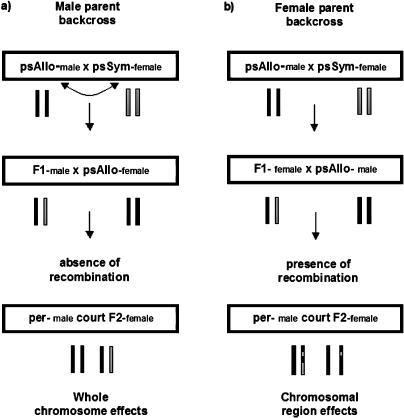
Experimental Design to Substitute Chromosomes or Chromosomal Regions Derived from Sympatry into an Allopatric Background and Measure Their Effect on Mating Discrimination F_1_ male-parent backcrosses (A) allow measurements of whole chromosome effects, while F_1_ female-parent backcrosses (B) measure specific chromosomal region effects. Curved arrow represents the reciprocal backcross of the one shown.

We investigated within-species variability in reinforced discrimination by estimating the chromosomal contributions to mating discrimination between two pairs of D. pseudoobscura populations. In each case, we performed a male-parent backcross in which a mixture of whole chromosomes from sympatry and allopatry (F_1_ genome) was substituted into an allopatric background (F_2_ backcross genome) (see Figure 1, left panel). Each male-parent backcross was also replicated with the reciprocal F_1_ cross between parental strains, thus ruling out any maternal effects and providing insight into the effect of the X chromosome on mating discrimination.

**Figure 2:**
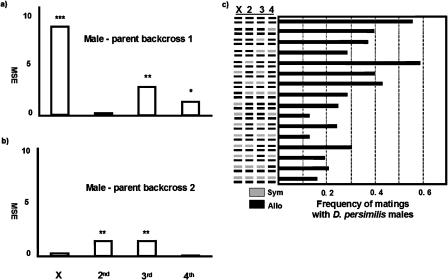
Mean Square Chromosomal Effects on Mating Discrimination (A) Male-parent backcross 1 shows the effects of substituting chromosomes derived from Mather, California (sympatry), into a background derived from Flagstaff, Arizona (allopatry). (B) Male-parent backcross 2 shows the effects of substituting chromosomes derived from Mt. St. Helena, California (sympatry), into a background derived from Mesa Verde, Colorado (allopatry). *, *p* < 0.005; **, *p* < 0.001; ***, *p* < 0.0001. (C) Combined chromosomal contributions to female mating discrimination. Small bars on the left represent chromosomes (X, 2, 3, and 4), while long bars on the right show the frequency of matings of backcross females with D. persimilis.

Our two backcrosses identified different chromosomes as affecting reinforced mating discrimination (binomial test of proportions for effects of all chromosomes, *p* < 0.01). For example, sympatric X and fourth chromosomes derived from Mather, California (male-parent backcross 1), contribute significantly to reinforced mating discrimination (*p* < 0.0001 for X chromosome, *p* < 0.005 for fourth chromosome, *n* of approximately 1,000 for all markers), while the same chromosomes show no detectable effect on reinforced mating discrimination when derived from Mt. St. Helena, California (male-parent backcross 2, *p* = 0.2297, *n* = 600 for all markers) (see Figure 2A and 2B). In contrast, the second chromosome shows the opposite relationship between the two backcrosses. The third chromosome shows marked effects on reinforced mating discrimination in both backcrosses, although at this level of resolution, it is impossible to tell whether this chromosome carries the same alleles in both sympatric populations. Figure 2C shows the genome composition of backcross females between Flagstaff, Arizona (allopatric), and Mather, California (sympatric), and their respective frequency of matings with D. persimilis males. The strongest effect is observed when both sympatric X and fourth chromosomes are substituted in an allopatric background, and no significant epistatic interactions were detected between chromosomes.

These results suggest that different alleles for reinforced mating discrimination are segregating within sympatric populations of D. pseudoobscura despite extensive gene flow within and between populations (Schaeffer and Miller 1992; Noor et al. 2000).

### Fine-Mapping the Genes Causing Reinforcement

**Figure 3:**
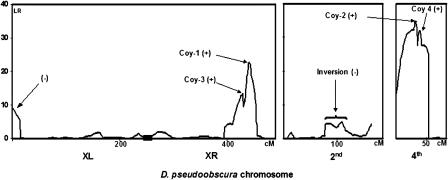
QTLs and Candidate Genes for Reinforced Mating Discrimination Each panel shows CIM estimations of chromosomal region effects on mating discrimination. Arrows point to major QTL locations and are named after their candidate genes. The direction of the chromosomal region effect on mating discrimination is shown in parentheses. The y-axis, LR, is the ratio of the likelihood value under the null hypothesis of no QTL to the likelihood value under the hypothesis that there is a QTL in a given interval of adjacent markers. The likelihood ratio significance threshold reflecting a Type I error of 0.05 is 11.5 s. The indicated inversion is a fixed chromosomal inversion differentiating D. pseudoobscura and *D. persimilis.*

We measured female mating discrimination against D. persimilis males in 1,500 F_2_ individuals derived from a female-parent backcross between a line derived from Mather, California (sympatric line), and a line derived from Flagstaff, Arizona (allopatric line), and genotyped 275 to 1,500 individuals for 70 markers dispersed along the four major chromosomes in D. pseudoobscura. Our initial single-marker analyses revealed significant associations between reinforced mating discrimination and three regions defined by markers located on the right and left arms of the X chromosome (XR and XL, respectively) (XR marker X021, *p* < 0.0001, *n* = 1,129; XL marker X002, *p* = 0.02, *n* = 1,293) and the fourth chromosome (4034 marker, *p* < 0.0001, *n =* 1,434). We were not able to detect an effect of any single region on the third chromosome even though nine markers were surveyed. Effects identified on XR and Chromosome 4 reinforced mating discrimination when the sympatric allele was present (positive), while the effect from XL was negative. After our initial scan, we used composite interval mapping (CIM) to account for any inflated estimates in the absence of background correction. In addition, several markers were genotyped around the X021 and 4034 regions with the goal of refining the segments containing the quantitative trait loci (QTLs). Figure 3 shows the major results from CIM, and confirms our previous observations: one major QTL was identified on XR around X021, and a suggestive one close to the telomere of XL. In addition, one major QTL was found on the fourth chromosome. These results validate our previous findings using male-parent backcross females and provide a high-resolution definition of regions contributing to reinforced mating discrimination.

#### X chromosome: Candidate genes *Coy-1* and*Coy-3*

A more careful examination of the X021 region showed that the QTL location (CIM LOD score = 5. 16), hereafter referred to as *Coy-1,* is estimated to lie between two additional markers, X021-A1 and X021-A4 (these markers are physically separated by 390 kb and by a recombination fraction of 4.5 centi-Morgans [cM]). According to the recently obtained genome sequence of *D. pseudoobscura,* there are seven genes between these two markers, one of which, *bru-3,* accounts for one-third of the sequence length of this region. In addition, CIM also identified another QTL, hereafter referred to as *Coy-3,* located between markers X021 and X021-B2, although with a weaker effect (CIM LOD score = 2. 84). There are approximately 200 kb and 30 genes between these markers and a recombination fraction of 3.5 cM. Finally, a third QTL was found near the XL telomere and, in contrast to the X021 region, showed a negative and weak additive effect (CIM LOD score = 2. 45). We tested this model for the X chromosome using multiple interval mapping and found that the strongest support is for *Coy-1,* followed by *Coy-3.* We were unable to recover any support for the QTL on XL. No epistatic interactions were detected among any QTLs.

#### Chromosome 4: Candidate genes*Coy-2* and*Coy4*

Dissection of the 4034 region using CIM split the effect into two QTLs for reinforced mating discrimination; we refer to them as *Coy-2* and *Coy-4,* respectively. These QTLs show the strongest effects (CIM LOD scores of 7.7 and 7, respectively). As with *Coy-1* and *Coy-3,* these QTL are additive and contribute positively to the degree of mating discrimination of F_2_ females. *Coy-2* is located next to marker 4034-A8. This marker is within a 300-kb region homologous to a D. melanogaster region containing a p-element insertion disrupting normal olfactory behavior (see Discussion for details) (Anholt et al. 2001, 2003). The D. melanogaster region contains 30 genes of which at least ten have known or predicted olfactory functions. The primary candidate gene for the disrupted olfactory behavior in the p-element mutant is *CG13982*. Interestingly, the D. melanogaster p-element mutation up-regulates expression of *bru-3,* suggesting a possible functional link between the candidate genes *Coy-1* and *Coy-2.* The second QTL in this region, *Coy-4,* is defined by two markers, 4003 and 4032, on each side of 4034. CIM places the QTL between 4003 and 4034, an approximately 200-kb region containing only nine genes. Five of these nine genes are a conglomerate of UDP-glycosyltransferases, genes preferentially expressed in the *Drosophila* antenna and coding for biotransformation enzymes involved in detoxification and olfaction (Wang et al. 2003). However, a more careful examination of the genes shows that their sequence overlap results from the inability of BLAST homology searches to distinguish the members of this gene family, suggesting that there may be only one or few UDP-glycosyltransferase genes here. Consequently, the number of candidate genes in the region may be reduced from nine to five genes, at least one of which is involved in olfaction. As before, we tested this model using multiple interval mapping and recovered significant support for *Coy-2* under stringent conditions and no evidence of significant epistasis among previously identified QTLs.

Based on these results and those for the X chromosome, we suggest that the strongest evidence for QTLs contributing to reinforced discrimination in sympatry lies with *Coy-1* and *Coy-2,* and that *Coy-3* and *Coy-4* are suggestive QTLs.

## Discussion

We have provided the first genetic dissection of an adaptive female preference involved in speciation by developing a QTL map for discrimination variation in Drosophila pseudoobscura. The resolution of our approach is novel to genetic studies of behavioral discrimination in that we have surveyed the genome with 70 microsatellite markers for chromosomal regions contributing to increased mating discrimination and have narrowed some of these regions to intervals containing as few as five genes. The role of these genes in reinforcing mating discrimination is supported by indirect evidence from D. melanogaster mutants: two of the major QTLs identified in our mapping experiments bear genes identified in smell impairment screenings of p-element mutants (Anholt et al. 2003). A gene in one of these intervals, *CG13982* (D. melanogaster Chromosome 2L), appears to up-regulate a second gene located in the other interval, *bru-3* (D. melanogaster X chromosome). Furthermore, we have shown that the chromosomal contributions to reinforced mating discrimination vary among strains of D. pseudoobscura. Finally, the chromosomal effects on mating discrimination are inherited in a dominant fashion, consistent with general theories on the evolution of adaptive characters (Haldane 1924). Below, we discuss these results in the context of several evolutionary hypotheses of reinforcement and speciation.

### The Genetics of “Basal” Versus “Reinforced” Female Mating Discrimination

Most genetic studies of female preference and sexual isolation have utilized between-species genetic crosses or non-hybridizing allopatric populations. Some of these studies suggest that female preference is a polygenic character (e.g., Moehring et al. 2004; Ting et al. 2001), while other researchers have found a very simple genetic basis for female discrimination (Doi et al. 2001). A study of another behavioral trait, response to odorants, showed that many genes contribute to olfaction, and epistasis plays a fundamental role in determining the specificity of odor identification (Anholt et al. 2001, 2003). We expect the genetics of reinforced female mating discrimination to bear some similarities to the genetics of overall female species preferences and/or traits involved in response to olfactory cues.

Available genetic data on “basal” female mating discrimination in D. pseudoobscura (between-species crosses using a D. pseudoobscura line derived from areas allopatric to D. persimilis) show that all QTLs for this trait map unequivocally to two inverted chromosomal regions separating it from D. persimilis (Noor et al. 2001a), one on XL and one on Chromosome 2. This result suggests that the regions we localized as contributing to reinforced mating discrimination (on XR and Chromosome 4) are distinct from those previously identified as contributing to basal discrimination. Hence, chromosomal inversions may have been crucial in allowing these species to persist in sympatry (Noor et al. 2001a), but the rearranged regions might not have contributed directly to the subsequent reinforcement of mating discrimination. This idea is consistent with data showing that a region *(DPS4003)* just 400 kb away from the QTL identified on the fourth chromosome seems to have introgressed recently between D. pseudoobscura and D. persimilis (Machado et al. 2002).

This result supports either a one-allele mechanism, perhaps controlling the genetics of variation within species for female mating discrimination if there was not strong assortative mating before sympatry, or possibly a two-allele mechanism, if reinforcement took place after sympatry and strong assortment had already evolved. The definitive test will be to determine whether introgressing the different D. pseudoobscura alleles into D. persimilis affects female discrimination in the same manner.

### Female Mating Discrimination Is a Composite Trait

**Figure 4:**
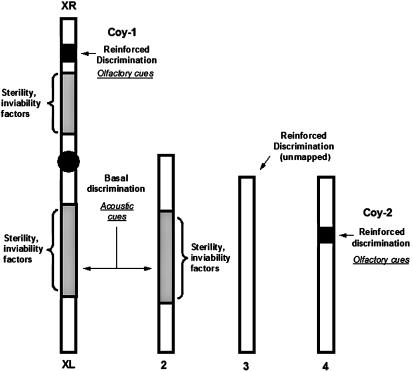
Genomic Distribution of Genetic Factors Preventing Gene Flow between D. pseudoobscura and D. persimilis Gray boxes denote fixed chromosomal inversions separating D. pseudoobscura and D. persimilis. Black boxes denote the genomic locations of QTLs for reinforced mating discrimination. Note that the third chromosome also conferrs high discrimination in sympatry, but no particular QTLs have been identified for this chromosome.

These “layers” of female discrimination (see Figure 4) are intimately related to the genetic differences being evaluated. Genes localized within fixed chromosomal regions inverted between D. pseudoobscura and D. persimilis are responsible for the first layer, basal discrimination. In contrast, the second layer, reinforced mating discrimination, is caused by genes localized outside those inverted regions. Basal discrimination appears to stem mostly from female responses to acoustic “courtship song” signal differences between D. pseudoobscura and D. persimilis. This is suggested by both a strong correlation in mating success of backcross hybrids with song parameters (Williams et al. 2001) and in playback experiments with wingless flies (M. Lineham, M. A. F. Noor, and M. Ritchie, unpublished data). Even though we cannot discard fine-tuning of the acoustic receiver signaling system in sympatric females, the nature of the candidate genes we identified suggests that olfactory responses might play a major role in the second layer of female preference. Non-auditory cues conferring reinforced discrimination are also suggested by behavioral data collected by Mark Lineham and Michael Ritchie (personal communication) showing that the rejection exercised by D. pseudoobscura females towards D. persimilis male song is the same in lines derived from sympatry and allopatry, even though females from the two regions clearly show differences in mating discrimination (Noor 1995; this study). Finding different genetic architectures for traits involved in speciation is expected under models based on selection on many traits (Rice and Hostert 1993). These traits may be a composite response of behavioral traits, as exemplified in this study, or ecology and behavior, as evidenced by Timema walking sticks, in which traits conferring ecological adaptation and traits contributing to mating discrimination act in conjunction to increase the overall level of sexual isolation between hybridizing populations (Nosil et al. 2003).

Our results suggest, albeit not conclusively, that reinforced mating discrimination is related to differences in response to olfactory cues. We have shown here that candidate regions on the fourth chromosome bear an unusual excess of olfactory genes, and some of these have been associated with specific olfactory responses in other *Drosophila*. Further, the fact that we mapped reinforced discrimination to two interacting gene regions involved in olfaction (bearing *bru-*3 and *CG13982*) was striking in this regard, supporting a potential role of olfactory response in reinforcement in these species.

We also observed differences among strains in the genetic architecture of reinforced mating discrimination. Such variation in genetic control may be common when populations exchanging genes differ in phenotype because of selection. Multiple alleles from different loci may have increased in frequency because of selection for discrimination, and these alleles sometimes spread into allopatry or are replaced by the allopatric alleles in sympatry. When sampling from single lines, we capture only a fraction of the genetic variation in mating discrimination, and sometimes a high-discrimination allele is even sampled from allopatry (as we observed in the QTL on XL). This observation should be typical in many QTL mapping studies that utilize strains within species with extensive gene flow among populations, as in the many studies of D. melanogaster variation.

In brief, these results show that basal and reinforced discrimination are different, species discrimination in D. pseudoobscura is a composite trait, and there is genetic variation within species in reinforced mating discrimination.

### Reinforced Mating Discrimination Is Inherited As a Dominant Trait

Recessive adaptive mutations are often lost before selection can screen their effects on the phenotype. Conversely, adaptive mutations that are visible to selection in a heterozygous state will be available for selection even at very low frequencies. Therefore, we expect that most adaptive mutations reaching high frequencies in a population are dominant (Haldane 1924, but see Orr and Betancourt 2001). This process is commonly referred to as Haldane's sieve and predicts that alleles for mating discrimination that increase in frequency in response to selection should be dominant (Orr and Betancourt 2001). Our study shows that F_1_ female offspring of crosses between allopatric and sympatric populations of D. pseudoobscura are as reluctant to mate with D. persimilis males as are D. pseudoobscura females from sympatric populations. This result implies that high discrimination can be expressed in heterozygous individuals, suggesting a dominant basis for the phenotype. In contrast, “basal” female mating discrimination seems to be a recessive trait (F_1_ female hybrids from crosses between allopatric D. pseudoobscura individuals and D. persimilis do not discriminate against D. persimilis males [Noor et al. 2001a]). Taken together, these results are consistent with genetic differences between basal and reinforced female mating discrimination and with general predictions from Haldane's sieve theory.

### Conclusions

This is the first study to provide a detailed description of the genetic basis of speciation by reinforcement. We conclude that, in *D. pseudoobscura,* (1) high discrimination in sympatry is inherited in a dominant fashion, (2) there is within-species variability for high female mating discrimination as evidenced by the different genetic architectures recovered in the male-parent backcross experiments, (3) there are multiple genes, possibly involved in olfaction, contributing to enhanced female mating discrimination, (4) some candidate genes for reinforcement identified here have been previously identified in p-element mutant screenings for smell impairment in *D. melanogaster,* (5) the genetic architecture of basal female mating discrimination is different from that of reinforced mating discrimination, and (6) inversions seem to play no direct role in creating or maintaining the genetic differences directly responsible for increased female mating discrimination in sympatry. However, these inversions seem to play a crucial role, as evidenced by previous studies (Noor et al. 2001a), in maintaining the identity of hybridizing species and thus providing time for selection to reinforce their sexual isolation. These results have broad evolutionary implications, as discussed above, and open exciting new avenues of research to understand the genetics of an adaptive behavioral trait involved in speciation.

## Materials and Methods

### 

Our approach is based on measuring in one species the effects of substituting chromosomal segments from a highly discriminant genome into a less discriminant genome. In particular, we (1) test for within-species variation in the genetic architecture of female mating discrimination (F_1_ male-parent backcrosses), (2) identify chromosomal regions contributing to reinforced mating discrimination (F_1_ female-parent backcross) and compare them to regions conferring basal mating discrimination, and (3) provide a set of candidate genes for increased mating discrimination.

#### Fly rearing and lines

D. persimilis flies were collected in 1993 from Mt. St. Helena, California. D. pseudoobscura flies were collected from Mather, California (1997), Mt. St. Helena, California (1997), Flagstaff, Arizona (1993 and 1997), and Mesa Verde National Park, Colorado (2001). Isofemale lines were established by rearing the offspring of individual females previously mated in the wild. All lines were maintained under a constant regime of temperature (20 °C) and humidity (85%) in diurnal/nocturnal cycles of 12 h and reared on a mixture of agar, dextrose, and yeast.

#### Reinforced mating discrimination in sympatry

Pairs of D. pseudoobscura isofemale lines from each of two populations were crossed: Mather (1997) 52 × 10 (California, sympatric with D. persimilis) and Flagstaff (1997) 16 × 17 (Arizona, allopatric to D. persimilis), respectively. Virgin F_1_ females from these crosses as well as D. persimilis males were routinely collected during afternoons and confined for 8 d. On the morning of the eighth day, individual females were confined with individual D. persimilis males. The rationale of this no-choice design is based on behavioral observations suggesting that females tend to copulate more often in the presence of single males than when multiple males approach them (Noor, unpublished data). Therefore, no-choice experiments should provide a more conducive setting for mating. The flies were observed for 10 min. If the male attempted fewer than three copulations, the pair was not scored, and the data were discarded. Otherwise, the pair was scored for successful copulation versus not (the male must have been on the back of the female for at least 1 min—the average copulation duration in D. pseudoobscura is 3 min). These protocols are the same used in Noor et al. (2001a). We performed Fisher exact tests to evaluate differences among D. pseudoobscura lines sympatric versus allopatric to D. persimilis. Comparisons between allopatric and sympatric populations were performed both for outbred lines and inbred lines and only between lines that were tested for the phenotype at the same time, thus controlling for environmental error. Our comparisons between the two allopatric lines and between the two sympatric lines were not temporally controlled, and therefore may have been subject to some environmental heterogeneity. We used pairs of D. pseudoobscura inbred lines that significantly differed in their degree of female mating discrimination against D. persimilis in our mapping experiments (see below).

#### The heritable basis of increased mating discrimination in sympatry

We measured the frequency of matings with D. persimilis males of F_1_ females resulting from crosses between sympatric and allopatric D. pseudoobscura lines. If F_1_ females discriminated as strongly as the parent derived from sympatry, then we concluded that higher (reinforced) mating discrimination was inherited as a dominant trait. Fisher exact tests where performed to evaluate this hypothesis (see Table 1).

#### Testing for male discrimination

D. persimilis males were tested against D. pseudoobscura females from the Mather 17 and Flagstaff 1993 strains. We measured the time to first attempted copulation, the number of attempted copulations, and the time between the first attempt to copulate and copulation itself. Analysis of variance was conducted to test for a difference between treatments.

#### Mapping approach

Microsatellite markers include those reported previously (Noor et al. 2000) and 100 more that were developed by scanning contig sequences produced by the D. pseudoobscura genome project (Richards et al. 2004). Microsatellites were tested for fixed allelic differences between D. pseudoobscura lines Mather 17 and Flagstaff 1993. All primer information, both for informative and non-informative markers, will be published elsewhere and is available upon request. A recombinational map with an average distance of 15 cM between markers was produced using the female-parent backcross (see below) and the multipoint-linkage approach implemented in MapMaker version 3.0 (Lander et al. 1987).

#### Male-parent backcross

Two male-parent backcrosses (*n*_1_ = 900 and *n*_2_ = 600 flies) were used to determine the chromosomal basis of reinforced mating discrimination and its natural within-species variation. Crossing over does not occur in male *Drosophila,* and they thus transfer whole chromosomes to their offspring (see Figure 1). Each F_1_ backcross female was scored for mating (as above), and its DNA was subsequently extracted. Lines used in each backcross were: for backcross 1, Mather (California) 17 and Flagstaff (Arizona) 1993, and for backcross 2, Mt. St. Helena (California) 7 and Mesa Verde (Colorado) 17. We consider strains derived from California as sympatric and strains derived from Arizona or Colorado as allopatric to D. persimilis.

We used microsatellite markers to score the origin of each chromosomal segment in backcross hybrid females. To determine the chromosomal contributions from each chromosome, we performed analyses of variance in which the dependent variable was mating discrimination and the independent variables the origin of each chromosome.

#### Female-parent backcross

Once we determined the chromosomal effects and their variation for mating discrimination, we scored an additional 1,500 females derived by backcrossing Mather 17 × Flagstaff 1993 F_1_ females to Flagstaff 1993 males (see Figure 1) for mating discrimination against D. persimilis. A total of 288 females were genotyped for all markers, and both single-marker analyses and CIM (Zeng et al. 1999) were used to identify QTLs contributing to reinforced mating discrimination. Both approaches consistently identified the same regions. An additional 1,200 females were genotyped for markers showing significant effects on mating discrimination. When implementing CIM, forward-backward stepwise regressions were used to search for target QTLs over 2-cM intervals while simultaneously fitting partial regression coefficients for background markers with a window size of 15 cM. We tested for epistatic interactions between significant QTLs using multiple interval mapping (Zeng et al. 1999). In all cases, procedures were carried out as implemented in QTL Cartographer (Basten et al. 1999). Significance threshold values were obtained by permutation analysis as described by Doerge and Churchill (Basten et al. 1996).

## Supporting Information

### Accession Numbers

The Flybase (flybase.bio.indiana.edu) accession numbers for the genes discussed in this paper are *bru-3* (FBgn0036379) and *CG13982* (FBgn0031811).

## Abbreviations

CIM: composite interval mapping
cM: centi-Morgan
QTL: quantitative trait locus
XL: left arm of the X chromosome
XR: right arm of the X chromosome
